# Effectiveness of pediatric integrative manual therapy in cervical movement limitation in infants with positional plagiocephaly: a randomized controlled trial

**DOI:** 10.1186/s13052-021-00995-9

**Published:** 2021-02-25

**Authors:** Iñaki Pastor-Pons, César Hidalgo-García, María Orosia Lucha-López, Marta Barrau-Lalmolda, Iñaki Rodes-Pastor, Ángel Luis Rodríguez-Fernández, José Miguel Tricás-Moreno

**Affiliations:** 1 Departamento de Fisiatría y Enfermería, Unidad de Investigación en Fisioterapia, Facultad de Ciencias de la Salud, Universidad de Zaragoza, Domingo Miral, s/n, 50009 Zaragoza, Spain; 2 Instituto de Terapias Integrativas, San Miguel, 16, 50001 Zaragoza, Spain; 3 Departamento de Fisioterapia, Facultad de Medicina, Universidad San Pablo CEU, Urbanización Montepríncipe, 28925 Alcorcón, Madrid Spain

**Keywords:** Positional plagiocephaly, Deformational plagiocephaly, Manual therapy, Physical therapy, Congenital muscular torticollis

## Abstract

**Background:**

Positional plagiocephaly (PP) is a cranial deformation frequent amongst children and consisting in a flattened and asymmetrical head shape. PP is associated with excessive time in supine and with congenital muscular torticollis (CMT). Few studies have evaluated the efficiency of a manual therapy approach in PP. The purpose of this parallel randomized controlled trial is to compare the effectiveness of adding a manual therapy approach to a caregiver education program focusing on active rotation range of motion (AROM) and neuromotor development in a PP pediatric sample.

**Methods:**

Thirty-four children with PP and less than 28 week-old were randomly distributed into two groups. AROM and neuromotor development with Alberta Infant Motor Scale (AIMS) were measured. The evaluation was performed by an examiner, blinded to the randomization of the subjects. A pediatric integrative manual therapy (PIMT) group received 10-sessions involving manual therapy and a caregiver education program. Manual therapy was addressed to the upper cervical spine to mobilize the occiput, atlas and axis. The caregiver educational program consisted in exercises to reduce the positional preference and to stimulate motor development. The control group received the caregiver education program exclusively. To compare intervention effectiveness across the groups, improvement indexes of AROM and AIMS were calculated using the difference of the final measurement values minus the baseline measurement values. If the distribution was normal, the improvement indexes were compared using the Student t-test for independent samples; if not, the Mann-Whitney U test was used. The effect size of the interventions was calculated using Cohen’s d.

**Results:**

All randomized subjects were analysed. After the intervention, the PIMT group showed a significantly higher increase in rotation (29.68 ± 18.41°) than the control group (6.13 ± 17.69°) (*p* = 0.001). Both groups improved the neuromotor development but no statistically significant differences were found. No harm was reported during the study.

**Conclusion:**

The PIMT intervention program was more effective in increasing AROM than using only a caregiver education program.

The study has been retrospectively registered at clinicaltrials.gov, with identification number NCT03659032. Registration date: September 1, 2018.

## Background

Asymmetries in the shape of the head are very frequent in typical and healthy newborns [1]. Positional plagiocephaly (PP) is a condition in which the head, and sometimes also the face of the baby are deformed as a result of pre- and/or post-birth external molding forces exerted on a malleable and growing cranium [2]. PP is generally characterised by an abnormally flattened and particularly asymmetrical shape of the cranium [3]. PP can be the result of pre-birth external forces (due to the way the child settles in the mother’s pelvis) as well as post-birth external forces (due to excessive time lying in supine) [4]. After the recommendation by the American Academy of Pediatrics to let children lie in supine position, a noticeable reduction (up to a 50%) of the sudden death syndrome was found [5]. However, an increase in nonsynostosic PP was also observed [6, 7]. While prevalence data are limited and depend on the geographical area, the highest estimations place the incidence of PP between 20 and 30% [8].

Children with PP are more likely to develop a number of conditions such as postural compensations [9], muscle flexibility and balance alterations [10], visual dysfunctions [11], temporomandibular dysfunctions [12], mandibular and occlusal asymmetries [13], neurodevelopmental alterations [14, 15], lower cognitive and academic results [16] and language adquisition deficit [17]. In addition, the literature has shown that congenital torticollis is the disorder which is most frequently associated to plagiocephaly [18, 19] and that the consequent restriction of cervical spine rotation to one side is associated to the positional preference of the head during lying [20]. Rogers et al. (2009) showed there was a restriction of active cervical rotation in children in almost all the cases of plagiocephaly studied, even without a previous diagnosis of congenital muscular torticollis (CMT) [21].

Some restriction of active cervical rotation, at least to one side, seems to be present in almost all PP cases. According to Hautopp et al. [22], when this restriction to one of the sides reaches an asymmetry greater than 15° CMT is diagnosed. Years later, Kaplan et al. used time of detection and amount of restriction in head rotation to classify different types of CMT. According to this classification, a restriction of less than 15° or a preferential position is considered as grade I CMT when CMT is detected before 6 months of age [23].

Nevertheless, the biomechanical factors that contribute to the restriction of cervical rotation AROM of the child, and later to CMT and PP are not fully understood. Moreover, the etiology of these conditions is not entirely clear and it is associated with multiple factors including delivery traumatisms [24], pre- and peri-birth compartment syndrome [1], alterations in the development of the sternocleidomastoid muscle [25] causing its stiffness and restriction of motion [26] and joint dysfunctions [27–34].

CMT treatment consists mainly in stretching exercises applied by the physical therapist or the family and in the stimulation to orientate the head towards the restriction side. Few studies have evaluated the effect of manual therapy on this cervical dysfunction. Moreover, the quality of these studies does not allow us to extract conclusions on its effectiveness [35].

The objective of this randomized controlled trial was to analyze the effect of manual therapy on the active cervical rotation and in the neuromotor development in a sample of children with PP. For that reason, we used an integrative approach based on manual therapy tailored for pediatrics, and sensory-motor stimulation included in a caregiver education program. Our secondary objective was to perform a reliability study of the cervical rotation AROM tests.

## Materials and methods

### Study design

A prospective randomized controlled trial has been conducted.

### Subjects

A sample of 34 patients younger than 28 weeks and diagnosed (by pediatricians) with non synostosic positional plagiocephaly, with a difference of at least 5 mm between cranial diagonal diameters [36], that is, infants with moderate or severe PP [37], were recruited from several Health Centers in Zaragoza (Spain). Genetic, metabollic or neurological diseases and severe neurodevelopment deficit were considered as exclusion criteria.

For the calculation of the sample size, we used non-published data from a previous pilot study with 41 subjects with similar characteristics and receiving a manual therapy intervention similar to the one used in the present study. An increase of 40° ± 17.5 in the overall cervical rotation AROM was obtained in this pilot study. The sample size was calculated using the GRANMO calculator (https://www.imim.cat/ofertadeserveis/software-public/granmo/), with the selection of two independent population means, bilateral contrast, with a α risk of 0.05, a ß risk of 0.20 and a ratio of 1 of the number of subjects between the groups. A minimal number of 4 subjects per group was obtained.

The subjects were randomized into 2 intervention groups with a final number of 17 subjects per group. The randomization design was generated using the on-line computer application at www.random.org/sequences. The evaluators were not told about this design.

For the reliability study of the measurement of the cervical range of motion, the data obtained previously in 44 subjects with the same characteristics were used. The total of 44 subjects in the sample was estimated as sufficient to guarantee a good or almost perfect degree of agreement [38].

The study has been registered at clinicaltrials.gov, with identification number NCT03659032.

An informative document about the study was provided to the parents and an informed consent was signed after they had read the document and their questions about the study had been answered. Regulations and guidelines regarding freedom, absence of coercion, disclosure of economic interests, understandable and complete information, confidentiality and acceptance were followed [39]. The study was approved by the Ethics Committee at the Aragon Health Sciences Institute (Registry No. C.P. - C.I. PI16/0275).

### Measurements

Clinical and demographic data were extracted from the medical history and the information provided by the parents: age (weeks), birth weight (gr), sex, premature, instrumental delivery, firstborn, multiple birth, positional head preference, plagiocephaly side, transport type, time in prone position with 1 month (min) and time in prone position with 2 months (min).

The following anthropometric values were measured with a caliper to assess the degree of cranial deformation: maximal cranial length (MCL), maximal cranial width (MCW) and the cranial diagonal diameters (CDD). Operating with these anthropometric values, the cranial index (CI) (cranial width÷cranial length× 100), the cranial vault asymmetry (CVA) (Long cranial diagonal diameter – Short cranial diagonal diameter) and the cranial vault asymmetry index (CVAI) ([Long cranial diagonal diameter – Short cranial diagonal diameter]/Short cranial diagonal diameter× 100) were calculated.

**Fig. 1:**
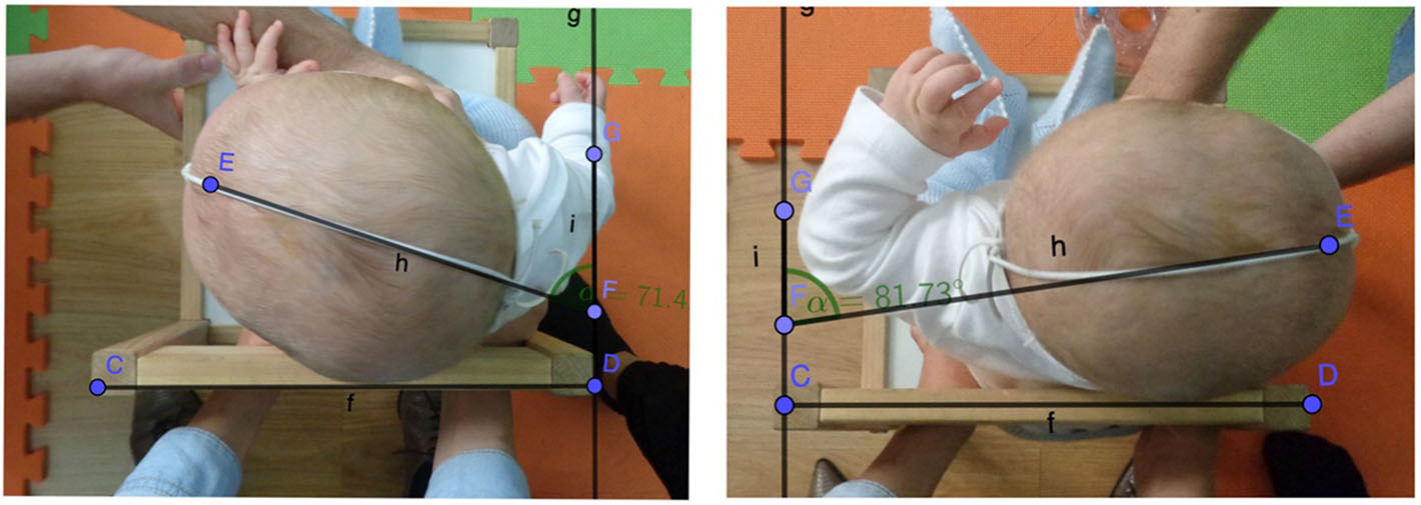
Coronal photographic view for the measurement of the cervical left and right rotation AROM with GeoCebra v.6

The dependent variables in this study were active rotation of the cervical spine and neuromotor development. The cervical rotation AROM was measured in each direction and was calculated considering the center of the neck as the axis of rotation. The final angle of movement was measured with respect to a line marked on the chair used by the patient. To optimize the evaluation, a cloth halo with a filter strap was used joining the most anterior part corresponding to the level of the nose and the most posterior one (Fig. 1). Although Murgia et al. used supine lying for this measurement [40], an upright position was chosen for the measurement in order to be able to observe the control of the head in the sagittal plane.

The subjects were sat in a low chair with their upper trunk held under the shoulders by one of their parents. An examiner stood in front of the child and stimulated them moving a sound toy in a semicircle around the child to provoke the rotation of the head until they reached the limit in each direction. Three valid repetitions to each side, in which the movement was not prematurely interrupted, were performed to ensure the maximal rotation. The cervical AROM to each side was registered by a photographic image from above [41].

Two photographic sessions were held before the intervention: one on the first day (first measurement) and the other 24 h later (second measurement). A third photographic measurement of the cervical rotation AROM was performed after the intervention. The examiner selected the photographies that showed the greatest head rotation. Those images were then analyzed with the program GeoCebra v.6 in order to measure cervical rotation AROM (Fig. 1). The analysis of the images was performed by an examiner, blinded to the randomization of the subjects within the study.

The neuromotor development was tested with the Alberta Infant Motor Scale (AIMS) [42], used in previous studies with similar samples [43]. AIMS is based on the measurement of motor infant performance on motor milestones from newly born to independent walking and it has been validated into Spanish [44]. The AIMS includes 58 items divided in 4 subscales: prone position (21 items), supine position (9 items), sitting (12 items) and standing (16 items). Each observed item in the motor infant performance scores 1 point. The sum is the infant’s total score. The total score and the age determine the percentile rank.

### Intervention

Subjects were randomly allocated to two groups. The interventions were administered in the Instituto de Terapias Integrativas de Zaragoza.

The control group received an evidence-based educational physical therapy program for the caregivers. This caregiver educational program consists in exercises to reduce the positional preference and to stimulate motor development, by advising parents on positioning, baby management and care [45–47]. This protocol was reinforced with an informative booklet about basic recommendations. The control group was convened once during the 10 weeks to control their evolution, listen to their difficulties, solve their questions and insist on the importance of the program of stimulation and positional advice.

The intervention group received the same educational approach as the control group and a specific protocol based on manual therapy tailored for pediatrics, an integrative concept of treatment that will be identified in the manuscript as pediatric integrative manual therapy (PIMT). The manual therapy protocol was applied by several pediatric physical therapists with specialized training and 4 years of experience and it included manual therapy techniques for the upper cervical spine and for remodeling the cranial deformation. The techniques used for remodeling the cranial deformation and their results are included in another manuscript pending publication.

**Fig. 2:**
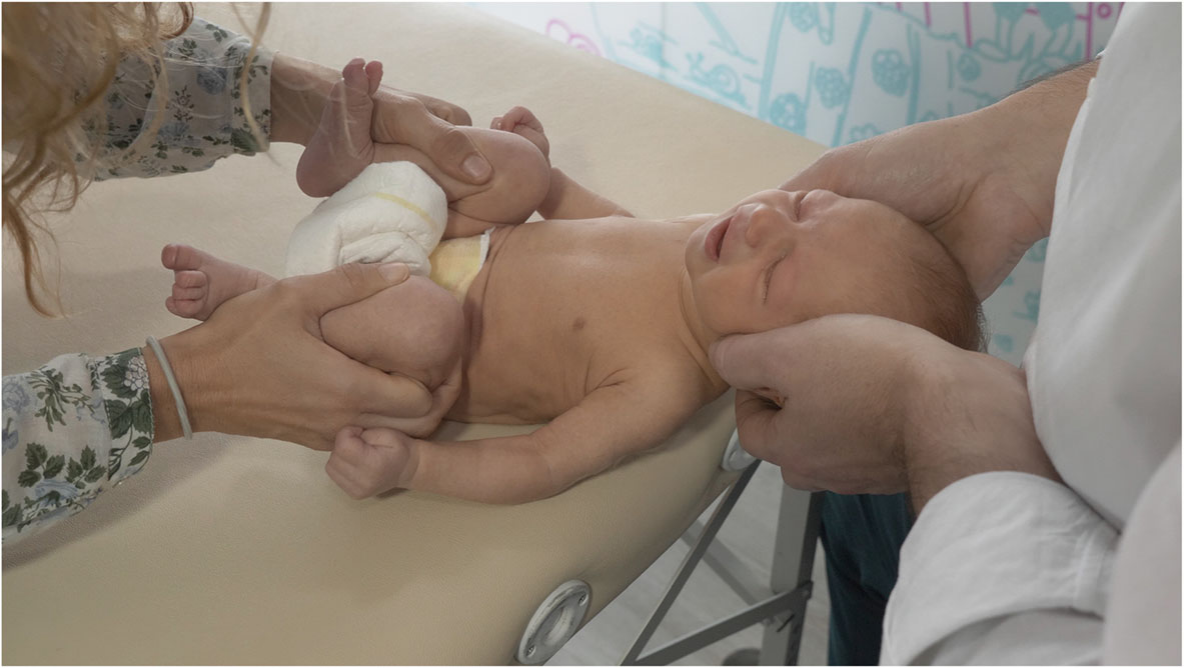
Upper cervical treatment technique from a flexion position

The objective of the manual therapy protocol for the upper cervical spine was to mobilize the occiput, atlas and axis to restore ROM. The technique applied consists in letting the baby’s head rest on the hands of the practitioner. Both fourth and fifth fingers were placed on the condylar area of the occipital bone, the middle finger on the articular processes of the axis, the index fingers on the articular processes of the cervical vertebrae below C2. The thumbs were placed on the anterior side of the transverse processes of the atlas to cause a very gentle dorsal positioning of the atlas. The practitioner applied a myofascial induction aiming to relax the cervical myofascial structures with a gentle traction while gently assisting head movements of flexion and extension, sidebending and rotation following the active and spontaneous movements of the baby [48] (Fig. 2). In all cases end-range positioning into cervical extension and rotation were avoided, following the recommendations of the International Federation of Orthopedic Manual Physical Therapists (IFOMPT) [49].

The subjects from the PIMT group were treated during 20-min sessions once a week for 10 weeks.

### Statistical analysis

The Kolmogorov-Smirnov test with the Lilliefors correction was used to test the normality of the distribution of the quantitative variables; the Shapiro-Wilk test was used for this purpose if *n* < 30. For the reliability analysis of cervical rotation measurements to both sides, Bland-Altman plots and analyses were used.

A descriptive analysis of the qualitative variables was carried out, offering the absolute and relative frequencies, as well as a descriptive analysis of the quantitative variables. The mean ± standard deviation was calculated when the distribution of the variables was normal and the median (Q1; Q3) values, where Q1 refers to value in 25th percentile and Q3 to value in 75th percentile, when the distribution was not normal.

The initial comparative analysis of the clinical and demographic qualitative variables was carried out with the Chi Square test, while the Fisher exact test was used when the table presented a box with an expected frequency lower than 5.

If the distribution was normal, the Student t-test for independent samples was used for intergroup comparisons of the quantitative pre-intervention variables. The Mann-Whitney U test was employed for these comparisons when the distribution was not normal. To compare intervention effectiveness across the groups, we calculated the improvement indexes of the dependent variables using the difference of the final measurement values minus the baseline measurement values (measurement 3-measurement 1). If the distribution was normal, the improvement indexes were compared using the Student t-test for independent samples; if not, the Mann-Whitney U test was used. The effect size of the interventions evaluated was calculated using Cohen’s d.

A one way ANCOVA was performed with any variable with differences in the initial moment as covariate, the group as factor, and the improvement index in the variable as dependent variable.

A confidence interval of 95% was established for the analysis. Statistical significance was set at *p* < 0.05. The statistical study was performed following the principles of intention-to-treat analysis, without attributing values in the second assessment to the subjects lost to follow-up.

## Results

### Reliability study

**Fig. 3:**
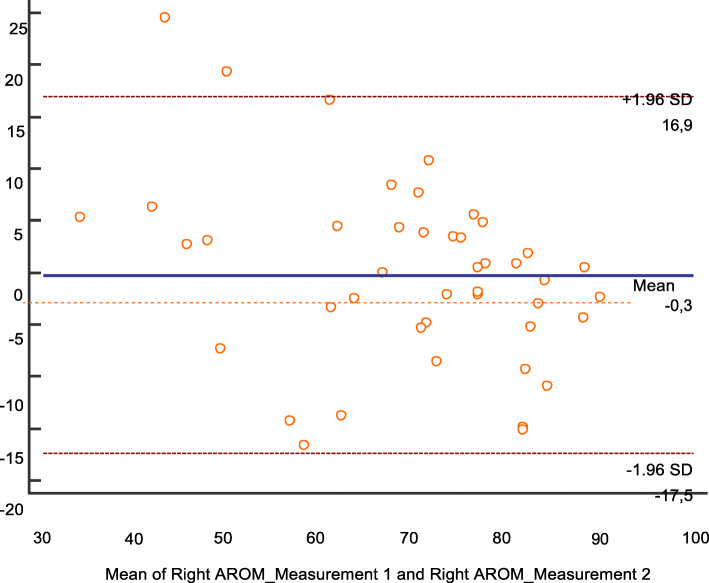
Bland-Altman plot for Right rotation AROM

We observed a difference of 3.8° in the cervical right rotation AROM between measurement 1 and 2 (Table 1). As the first measurement did not show a normal distribution, the intraclass correlation index was not recommended and a Bland-Altman plot (Fig. 3) was used to evaluate reliability. In the plot, a mean difference (mean value in measurement 1 – mean value in measurement 2) of − 0.3, close to 0, was observed so the systematic measurement error was low.

**Table 1:** Descriptive analysis of the AROM repetitive measurements before intervention; ^a^ Mean ± standard deviation; ^b^ Median (Q1; Q3); *p value < 0.05

Infants with PP (*n* = 41)		Descriptives	Kolmogorov-Smirnov Sig.
Right AROM (°)	Measurement 1	73.5 (60.2; 78.5) ^b^	0.027*
	Measurement 2	69.7 ± 15.9 ^a^	0.064
Left AROM (°)	Measurement 1	65.1 (58.1; 70.7) ^b^	0.014*
	Measurement 2	66.6 (58.8; 70.9) ^b^	0.01*

Moreover, the difference between the measured value in the measurement 1 and 2 exceeded the established agreement limit of two standard deviations only in two subjects (4.6%), so the concordance was adequate.

**Fig. 4:**
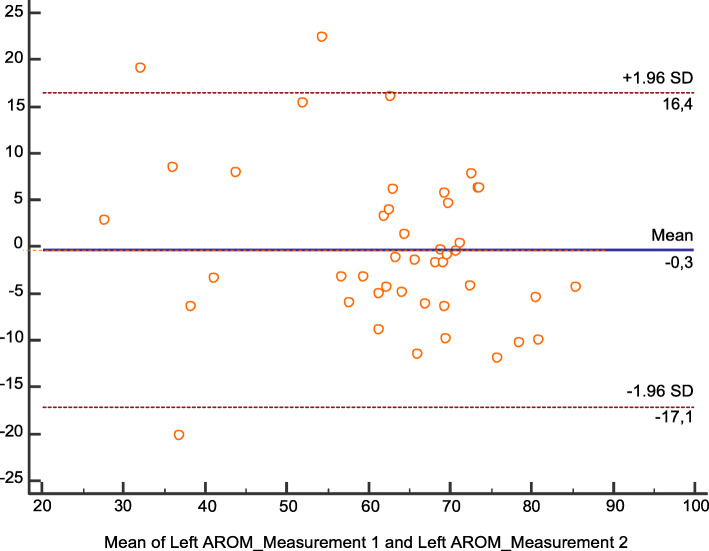
Bland-Altman plot for Left rotation AROM

We observed a difference of 1.5° in the cervical left rotation AROM between measurement 1 and 2 (Table 1). As none of the two measurements showed a normal distribution, the intraclass correlation index was not recommended and a Bland-Altman plot for the reliability evaluation (Fig. 4) was used. In the plot, a mean difference (mean value in measurement 1 – mean value in measurement 2) of − 0.3, close to 0, is observed so the systematic measurement error is low.

The difference between the measured value in the measurements 1 and 2 exceeded the established agreement limit of two standard deviations only in three subjects (6.8%), so the concordance was adequate.

### Intervention study

A total of 34 subjects were included in the study. Seventeen were assigned to the PIMT intervention group and 17, to the control group (just a caregiver education program). Two subjects were lost in the intervention group, so the final analysis comprised 15 subjects in the intervention group and 17 in the control group. Demographic characteristics were comparable in the two groups (Tables 2 and 3). Anthropometric measurements and head shape were comparable in both groups although right AROM was more restricted in the PIMT group in the baseline evaluation (Table 3).

**Table 2:** A comparative descriptive analysis of the qualitative variables in the baseline examination. PIMT (Pediatric Integrative Manual Therapy). ^a^Statistical analysis using the Chi Square test; ^b^statistical analysis performed with the Fisher exact test

Baseline examination				
Qualitative variables	Infants with PP (*n* = 34)	PIMT Group (*n* = 17)	Control Group (*n* = 17)	*p* value
Sex ^a^				
Women	47.1%	52.9%	41.2%	0.492
Men	52.9%	47.1%	58.8%	
Premature ^a^	18%	29.4%	5.9%	0.175
Instrumental delivery ^b^	20.6%	17.6%	23.5%	1.000
Firstborn ^a^	70.6%	70.6%	70.6%	1.000
Multiple birth ^b^	20.6%	23.5%	17.6%	1.000
Positional head preference ^b^	94.1%	100%	88.2%	0.485
Plagiocephaly side ^a^				
Right	64.7%	52.9%	76.5%	0.151
Left	35.3%	47.1%	23.5%	
Transport type ^b^				
Trolley	97.1%	100%	94.1%	1.000
Babies backpack	2.9%	0%	5.9%

**Table 3:** Homogeneity of the quantitative variables in the baseline examination. PIMT (Pediatric Integrative Manual Therapy). MCC (Maximum Cranial Circumference). CI: (Cranial Index). CVA: (Cranial Vault Asymmetry). CVAI: (Cranial Vault Asymmetry Index). AROM: (Active Range of Movement). AIMS (Alberta Infant Motor Scale). ^a^ Statistical analysis using the Student t-test. ^b^ statistical analysis using the Mann-Whitney U test; * significant *p* value

Baseline examination				
Quantitative variables	Infants with PP (n = 34)	PIMT Group (n = 17)	Control Group (n = 17)	p value
Age (weeks) ^b^	17.2 ± 4.3	17.3 ± 4.3	17.2 ± 4.6	0.938
Birth weight ^a^ (gr)	3114 ± 544.7	3040 ± 605.3	3188 ± 483.7	0.437
Time in prone position with 1 month ^b^ (min)	2 (0; 5.3)	1 (0; 5)	5 (5; 16)	0.520
Time in prone position with 2 months ^b^ (min)	5 (1; 10)	2 (0.5; 10)	10 (5; 11)	0.228
CVA ^b^ (mm)	7.83 (6.29; 9.75)	8.20 (6.50; 11.75)	7.00 (5.83; 9.33)	0.196
CI ^a^ (%)	87.79 ± 6.71	88.35 ± 6.39	87.04 ± 7.14	0.522
CVAI ^b^ (%)	6.04 (4.95; 8.01)	6.59 (5.20; 9.30)	5.37 (4.51; 7.86)	0.153
Right rotation AROM ^a^ (°)	71.5 ± 10.7	66.4 ± 11.7	76.5 ± 6.9	0.005*
Left rotation AROM ^b^ (°)	65.6 (59.7; 70.7)	68.4 (62.6; 72.8)	63.0 ± 10.6	0.459
Total rotation AROM ^b^ (°)	140.3 (129.5; 147.8)	134.0 (125.9; 145.9)	143.4 (133.5; 150.5)	0.163
AIMS ^b^ (%)	25 (10; 50)	25 (10; 50)	25 (10; 50)	0.683

### Cervical rotation AROM restriction

The increase of right rotation AROM was significantly larger in the PIMT group than in the control group, 13.4 ± 9.1° and − 1.6 ± 9.5° (*p* = 0.000) respectively (Table 4). Right AROM was lower at baseline in the intervention group. ANCOVA with right AROM at baseline as covariate, group as factor and improvement index in right AROM as dependent variable showed statistical significance for group (*p* = 0.026) and for right AROM at baseline (*p* = 0.000).

**Table 4:** Summary of the the variables with descriptive and comparative data on their Improvement Indices. PIMT: (Pediatric Integrative Manual Therapy). MCC: (Maximum Cranial Circumference). CVA: (Cranial Vault Asymmetry). CI: (Cranial Index). CVAI: (Cranial Vault Asymmetry Index). AROM: (Active Range of Movement). AIMS (Alberta Infant Motor Scale). Statistical analysis performed using the Student t-test for independent samples. ^a^ Mean ± standard deviation statistical analysis using the Student t-test; ^b^ median (Q1; Q3) statistical analysis using the Mann-Whitney U test; *significant *p* value

Descriptive and comparative of the Improvement Indices				
Variables	PIMT Group *n* = 15	Control Group n = 17	Sig.	Cohen’s d effect size
Right AROM ^a^ (°)	13.4 ± 9.1	−1.6 ± 9.5	0.000*	1.61
Left AROM ^a^ (°)	16.3 ± 11.7	7.7 ± 13.7	0.070	0.67
Total AROM ^a^ (°)	29.7 ± 18.4	6.1 ± 17.7	0.001*	1.3
AIMS ^b^ (%)	0 (0; 25)	0 (0; 25)	0.887	0.04

The increase of left rotation AROM in the PIMT group was not significantly different to the increase in the control group, 16.3 ± 11.7° and 7.7 ± 13.7° (*p* = 0.07) respectively (Table 4).

The total cervical rotation AROM increased in both groups. The improvement was significantly better in the PIMT group than in the control group, 29.7 ± 18.4° and 6.1 ± 17.7° (*p* = 0.001) respectively (Table 4).

### AIMS

There were no differences in the improvement between the groups in the AIMS (*p* = 0.887) (Table 4).

## Discussion

Reliability study showed low systematic measurement error and adequate concordance in right and left cervical rotation measurements.

After the intervention, the PIMT group showed a significantly higher increase in right cervical rotation AROM and total cervical rotation AROM. No significant differences have been obtained between groups in left cervical rotation AROM and AIMS.

### Reliability study

The photogrammetric measures were found to be reliable and the systematic error of measurement found was considerably lower than the changes generated with PIMT group.

Other authors using static photography to measure the position of the head in children with congenital torticollis obtained reliability values similar to those in our study [50].

Klackenberg et al. [51] showed a good reliability with static photography compared to goniometry in children with congenital torticollis for the measurement of passive cervical rotation in supine. These authors established a difference between measurements of ≤6° was clinically acceptable. Following Klackenberg et al. (2005), our study shows a clinically acceptable degree of agreement.

Different methodologies to evaluate active range of motion (AROM) [41, 52] and passive range of motion (PROM) [53, 54] have been described in the literature on cervical mobility in infants. Even though PROM is the most common assessment for children with CMT and PP, and has been described in detail in the literature, AROM assessment has also been used on babies with PP [40]. Despite being recommended, the procedure for AROM assessment has not been clearly described and no reliability studies have been carried out [53, 55].

To our knowledge, Murgia et al. [40] is the only study that has analyzed cervical rotation AROM. In spite of the measurement difficulties, these authors also showed a good reproductibility in babies with plagiocephaly, but their results are not comparable to ours because they used a qualitative scale for the level of dysfunction (neutral position; full ROM; mild limitation; moderate limitation; severe limitation) but did not include degrees of rotation. Furthermore, they did not use static photography [40]. A recent systematic review about the evaluation of the cervical ROM in babies with PP concluded that static photography improved measurement properties [56].

### Intervention study

Our sample consisted of 52.9% males and 47.1% females. The mean age was 17.2 ± 4.3 weeks, slightly lower than other studies [36, 43]. The average birth weight of the subjects in our sample was 3114 ± 544.7 g., which is within normal values. While 18% of the subjects were preterm, 20.6% had an instrumental delivery. These percentages were lower than those in other studies with similar samples. In addition, 70.6% of them were first-born.

The median time spent in prone during the first month of life was 2 (0; 5.3) minutes a day and 5 (1; 10) minutes during the second month, which are lower values than recommended [57].

Most of of the subjects (94.1%) showed a positional preference in the baseline measurement. 64.7% showed a flattening of the right posterior side and 35.3% in the posterior left side of the head. According to literature the right-left ratio in the posterior flattening is 2:1 in PP, probably due to the preferential position of head contact on the right side, associated to a higher range of motion into right cervical rotation and a restriction in left rotation in most of the newborns [58]. Our results also show a predominance of flattening on the right side, although not reaching 50%, and a greater limitation of movement into left cervical rotation has been observed in the entire sample and in the control group. In fact, in this group where left cervical rotation was clearly more limited than in the PIMT group, right posterior flattening percentage reached 76.5%.

Only 2.9% of the subjects were transported mainly in baby backpack compared to some 97.1% that used baby strollers. Therefore, we cannot study the possible influence of this variable in mobility restriction.

Mean CI was 87.79 ± 6.71 indicating a more brachiocephalic cranium. Newborns with a more brachiocephalic index have shown more restriction in cervical rotation PROM than dolicocephalic children with a lower CI [53].

Median CVA was 7.83 (6.29; 9.75) mm. According to Mortenson & Steinbok’s classification [37], the sample had a moderate PP (≤ 12 mm).

Median CVAI was 6.04 (4.95; 8.01) %. Plagiocephaly severity scale, pursuant to the Children’s Healthcare of Atlanta [59, 60] of our sample was level 2. The scale classifies plagiocephaly severity according with CVAI in five levels: level 1: within normal limits when CVAI is < 3.5; Level 2: mild severity when CVAI is 3.5 to 6.25; level 3: moderate severity when CVAI is 6.25 to 8.75; level 4: severe when CVAI is 8.75 to 11, and very severe as CVAI greater than 11.

The subjects in our sample obtained lower values than the reference values for cervical rotation. The total degress of cervical rotation AROM had a median of 140.3 (129.5; 147.8)°, 71.5 ± 10.7° to the right and 65.6 (59.7; 70.7)° to the left. However, Aarnivala et al. obtained an average of 189.1° in PROM in a sample of 155 healthy newborns [53]. It is generally accepted that a PROM of 110° to each direction is normal in healthy babies [25, 40]. However, there are insufficient references, especially for the cervical AROM. Using an inclinometer Arbogast et al. found that the right rotation AROM in children from 3 to 5 years old was 68.1° and 68.8° to the left. With videography, the results were 79.5° to the right and 81.9° to the left, 161.4° in total. In 6–8 years-old children, the rotation diminished to 78.8° to the right and 78.2° to the left. Rotation was slightly lower between 9 and 12 years old [61]. The results for 3-year-olds could be expected to show higher AROM. Nevertheless, there is no literature confirming this. Considering the results of Arbogast et al., we could assume that there was a restriction greater than 15° to the left and therefore, following Kaplan these subjects would be diagnosed as grade II torticollis, although Kaplan’s criteria were initially designed for rotation PROM [55].

PIMT group obtained a statistical significant increase compared to the control group in right rotation AROM. The measurement of the right rotation AROM at baseline in the PIMT group has contributed to the large effect size detected in this variable, greater than on the left rotation. Right rotation AROM was lower than in the control group and therefore it had a greater margin for improvement, although without invalidating the effect of the intervention, which is still significant in the ANCOVA analysis. There were no significant differences between both groups in left rotation AROM increases. PIMT group obtained a statistically significant increase compared to the control group in total cervical rotation AROM. The PIMT group even overcame the normal values described for the population of 3 year-olds in Arbogast et al. [61]. These results might be explained by the fact that PIMT protocol for the upper cervical spine mobilized the occiput, atlas and axis. Several studies have related joint dysfunction in the upper cervical spine related to CMT. Atlantoaxial rotatory fixation (AARF) [62] has been linked to torticollis [27]. Several imaging studies have also described a link between CMT and the C1-C2 joint subluxation, which causes C1 to be in a more ventral position, rotated or sidebent [30, 32]. In the sample for the study carried out by Sardhara et al. [32], 52% of the patients ranging from 5 to 64 years of age had evident clinical torticollis and an even higher percentage of the subjects with torticollis were associated with greater degrees of C1 displacement in all planes. Biedermann described an upper cervical dysfunction related to torticollis in newborns termed KISS-syndrome (Kinetic Imbalance due to suboccipital strain) [33]. The KISS-syndrome was described as a joint dysfunction of the occipital-vertebral junction that is typically treated with manipulation. However, no clinical trial has been developed to evaluate the effectiveness of his intervention. In addition, certain risks associated with this intervention need to be considered [34].

Keklicek and Uygur applied a protocol of soft tissue mobilization techniques to 10 babies with torticollis and compared it to a protocol of stimulation and stretching in the control group. At 6 weeks, cervical rotation PROM to the most restricted side had an average increase of 44.8° compared to an increase of 23.1° in the control group, supporting the addition of manual therapy in the short term. Both groups improved significantly. At 12-week and at 18-week follow up, both groups had a similar passive rotation, approximately 88°. These values are slightly greater than our values in the PIMT group, although Keklicek and Uygur measured the PROM, which limits the comparison of these results [63].

Regarding the motor development measured with the AIMS, both groups were at baseline within the normal percentile, as percentile 10 is identified as the limit for gross motor development delay. The change in motor skills, favoring both groups and without statistical differences between them, may be attributed to the stimulation of the motor development with the baby backpack, the stimulation with objects towards both directions and the increased time spent in prone position, as recommended within the common educational program for both groups. Considering these results, it seems that the restriction of cervical mobility and the plagiocephaly did not affect the neuromotor development in our sample, which does not agree with the findings of other studies [64, 65].

This study had several limitations. On the one hand, the literature has not studied the relationship between the cervical rotation AROM and PROM yet, limiting the comparison of these findings. Moreover, the control group could have included a muscle stretching or passive mobilization protocol, but our aim was to focus on the effects of following recommendations in the field of physical therapy regarding positioning and stimulation. Finally, it would have been useful to have follow up data to analyze long-term outcomes.

In spite of the described limitations, PIMT could be considered as an efficient therapeutic alternative for the treatment of restrictions of the cervical mobility in babies with plagiocephaly. The involvement of the upper cervical spine in the restrictions of the cervical rotation should be considered in this specific population. This manual approach would be a less dangerous and aggresive therapy than quick chiropractic manipulations [66] or surgery procedures [32] described in studies with samples probably sharing biomechanical conditions with our sample of study.

## Conclusions

Adding manual therapy to a caregiver educational program is associated with a better outcome in terms of neck movement in positional plagiocephaly.

No outcome differences in neuromotor development was shown by adding manual therapy to a caregiver educational program in positional plagiocephaly.

## Data Availability

The datasets used and/or analysed during the current study are available from the corresponding author on reasonable request. All data generated or analysed during this study are included in this published article.

## Abbreviations

AAP: American Academy of Pediatrics
AARF: Atlanto-axial rotatory fixation
AIMS: Alberta Infant Motor Scale
AROM: Active range of motion
CDD: Cranial diagonal diameters
CI: Cranial index
CMT: Congenital muscular torticollis
CVA: Cranial vault asymmetry
CVAI: Cranial vault asymmetry index
IFOMPT: International Federation of Orthopedic Manual Physical Therapists
KISS: Kinetic Imbalance due to suboccipital strain
MCL: Maximal cranial length
MCW: Maximal cranial width
PIMT: Pediatric integrative manual therapy
PP: Positional plagiocephaly
PROM: Passive range of motion
RCT: Randomized control trial
ROM: Range of movement
